# What can go wrong in hyperemesis gravidarum: Wernicke–Korsakoff syndrome in Bulawayo, Zimbabwe

**DOI:** 10.1002/ccr3.1462

**Published:** 2018-03-08

**Authors:** Fiona T. Nhari, Narcisious B. T. Dzvanga

**Affiliations:** ^1^ Department of Medicine United Bulawayo Hospitals 2 St Lukes Road, Ascot Bulawayo Zimbabwe

**Keywords:** Hyperemesis gravidarum, pregnancy, thiamine, Wernicke–Korsakoff

## Abstract

We report two cases of Wernicke–Korsakoff syndrome (WKS) secondary to hyperemesis gravidarum. Oral thiamine was administered (intravenous unavailable locally). However, both the patient's condition improved in the first 48 h and progressively over a month. Initially, WKS is missed due to rare feature of full classical triad of symptoms, especially in nonalcoholics.

## Introduction

Wernicke–Korsakoff syndrome (WKS) is due to a deficiency of vitamin B1 (thiamine). Thiamine is an important vitamin, which acts as a cofactor for several enzymes such as pyruvate dehydrogenase and transketolase, which are involved in the aerobic respiration. Our brain has a very high metabolic demand, and the energy for these metabolic processes taking place in the brain comes from the aerobic respiration. When the thiamine level in the body becomes inadequate, this energy‐producing pathway fails, resulting in the death of neural tissues and the subsequent appearance of the clinical features 1.

Wernicke–Korsakoff syndrome is characterized by a profound anterograde and retrograde amnesia, disorientation in place and time, and lack of insight. Peripheral neuropathy, nystagmus, and ataxia may also be present, the latter two signs being related to earlier Wernicke episodes. Other common signs and symptoms include confabulation, anxiety, and apathy 2. Wernicke encephalopathy (WE) is also caused by the thiamine deficiency and is characterized by a set of acute psychotic symptoms and ophthalmoplegia. This condition can be reversed by thiamine supplementation. But if untreated, Wernicke encephalopathy (WE) can progress into an irreversible stage called the WKS. Thus, they are two extremities of a spectrum of clinical manifestations. The key difference between WE and WKS is that WE is reversible, whereas WKS is irreversible 1.

The condition is usually associated with chronic alcoholism, but it should be considered in patients with anorexia nervosa, prolonged vomiting associated with chemotherapy, gastrointestinal disease, hemodialysis, and hyperemesis gravidarum. In a large literature review, the most frequent causes of WE in nonalcoholic patients were neoplastic disease (18.1%) and gastrointestinal surgery (16.8%) 3.

## Case Description

### Case 1

We present a 27‐year‐old female P0G1, GA 16+1/40 readmitted by gynecologists for hyperemesis gravidarum 1 day after discharge (on metoclopramide, pyridoxine, and magnesium trisilicate). She presented with more than eight episodes of intractable vomiting as well as blurred vision.

The problem had persisted for several weeks and she had been admitted previously for the same condition with minimum improvement. She had no comorbidities, never had any surgery, does not drink alcohol, is from a middle‐class family and had balanced diet with regular meals daily, and had only been on metoclopramide 10 mg po tds and pyridoxine 25 mg po od. This was her first pregnancy. However, there is a family history of hyperemesis gravidarum (mother and sister in all pregnancies).

On this particular admission, she was ill looking, had mild pallor, and was alert and no anomalies on physical examination or vitals. She was managed on promethazine, pyridoxine, normal saline, and co‐codamol. After a week, physicians were consulted, but after several events had occurred, she had an intrauterine death followed by dilatation and curettage She continued to vomit with no hematemesis, although down to three times a day, vision continued to decline, had pain in left hand, weakness of lower limbs, and was unable to mobilize. She had hypersalivation, mild confusion with confabulation that progressed to not communicating at all, and level of consciousness was Glasgow Coma scale of 7/15. On review by physicians, all were noted in addition to the above was horizontal nystagmus and pain on palpation of all muscle groups of both lower and upper limbs.

Several investigations were ordered during her 3 weeks in hospital: Tables 1 and 2.

HIV (negative), RPR (negative), rheumatoid factor (negative), RBS(6.2),urinalysis (blood ++, ketones++, and Alb 3), ANA (negative),muscle creatine kinase‐601 (upper limit 229), chest X‐ray‐normal, lumbar X‐ray‐normal.
Brain CT scan (no MRI machine at hospital): moderate brain atrophy, mild dilated temporal horn of lateral ventricle, and left sphenoid sinusitis.
Fundoscopy: RT and LT both: valsalva retinopathy with retinal hemorrhages, superior to disk, macula normal, and cup disk ratio 0.3.
Tests not carried out due to lack of consent or funds: lumbar puncture, BMA, serum folate, serum B12, rpt muscle CK, TFTs, and serum Mg.

**Table 1:** Weekly FBC

	Week 1	Week 2	Week 3
WBC	6.24	11.16	17.26
RBC	3.17	3.46	3.42
HB	9.1	10.9	10.9
MCV	86.2	89.9	104.7
PLT	31	157	644

**Table 2:** Weekly U&E

	Week 1	Week 2	Week 3
K	2.13	3.56	4.02
NA	139	143	138
Urea	3.07	3.50	4.55
Creatinine	21.3	50.6	51.5

WE made an impression of Wernicke–Korsakoff syndrome secondary to hyperemesis gravidarum, polymyositis, hypokalemia, peripheral neuropathy (dry beriberi), and megaloblastic anemia secondary to hyperemesis gravidarum. She was treated with oral thiamine 100 mg po od as iv was not available locally and even the oral was difficult to procure (within 48 h patient improved was alert, communicating, ocular signs resolved, but still had some retrograde and some anterograde amnesia). She also received iv potassium supplements, prophylactic heparin, amitriptyline, prednisolone, indomethacin, folate, and physiotherapy.

Her condition continued to improve. On discharge, she still had the retrograde/anterograde amnesia but the rest of her clinical picture had improved she was mobilizing with assistance and is pending review in outpatient with pending vit B12/BMA results.

### Case 2

A 21‐year‐old female P0G1 GA 14/40 was admitted as a referral to the physicians from a Rural Hospital with a history of hyperemesis gravidarum. She was said to have developed sudden confusion and irritability. On examination, she was confused, irritable, had horizontal nystagmus, ophthalmoplegia, no signs of meningism, PEARL, and a Glasgow Coma scale of 9/15.

Investigations were made as follows: obstetric ultrasound (GA 14/40, viable, single intrauterine fetus), CSF analysis (glucose‐3.0, protein 0.5, India Ink NEG), FBC (WBC 10.4, HB 11.5, MCV 78.1, and PLT 273), U&E (UREA 7.2, CREA 101, and K 3.17), LFTs (AST 57, ALT 48.3, ALP 74, and ALB 31), HIV (NEG). Brain CT/MRI (not performed due to lack of funds), chest X‐ray (right upper lobe cavitation consistent with pulmonary tuberculosis).

We made an impression of (1) Wernicke–Korsakoff syndrome secondary to hyperemesis gravidarum and (2) pulmonary tuberculosis. The patient was treated with oral thiamine 100 mg po OD (iv not available locally) and anti‐TB drugs 3FDC HRZE.

By day 3, ophthalmoplegia and nystagmus had improved although patient developed lower limb paralysis consistent with sensorimotor peripheral neuropathy. Day 7 patient developed quadriparesis, similar to ascending peripheral neuropathy. Day 9 repeat obstetric ultrasound showed intrauterine fetal death, and management of the patient was surgical evacuation of the uterus. Relatives then requested discharge back to rural center. We discharged patient on thiamine 100 mg po od and 3FDC HRZE.

Our final diagnosis on discharge was Wernicke–Korsakoff syndrome with central pontine myelinolysis and pulmonary tuberculosis. Several months later, patient is said to be improving.

## Discussion/Conclusion

Wernicke–Korsakoff syndrome in hyperemesis gravidarum does occur and maybe it is more common in our environment but under diagnosed as the above two patients were admitted a few months apart. Prompt treatment and a very high index of suspicion is key, although literature does recommend iv thiamine oral thiamine did show an improvement in the patient's condition within 48 h. Prophylactic thiamine as recommended by current gynecologists guidelines might be beneficial in patients with long‐standing hyperemesis gravidarum with protracted vomiting 4, 5, requiring fluids for more than 24 h, especially before administration of dextrose or parenteral nutrition 5, to prevent Wernicke–Korsakoff syndrome although availability of the thiamine whether oral/iv needs address in resource‐limited settings to avoid such sequel. WE is marked neuropathologically by lesions of periventricular nuclei, hypothalamic nuclei, tectal plate, and thalamus, which are caused by thiamine (vitamin B1) deficiency and results in WE's cardinal signs of ophthalmoplegia, nystagmus, ataxia, and confusional state (as noted in Victor et al. 6, 7) therefore, MRI scan is more beneficial than CT scan which we had at our facility in diagnosing WE; hence, we had no specific findings, the sensitivity of MRI in detecting WE was only 53% and the specificity was 93% 8.

## Conflict of Interest

None declared.

## Authorship

FTN: author. NBTD: guided the author in writing the manuscript and proofread the final manuscript.
